# Protective Effects of Costunolide Against D-Galactosamine and Lipopolysaccharide-Induced Acute Liver Injury in Mice

**DOI:** 10.3389/fphar.2018.01469

**Published:** 2018-12-20

**Authors:** Jingxin Mao, Man Yi, Rui Wang, Yuanshe Huang, Min Chen

**Affiliations:** Key Laboratory of Luminescent and Real-Time Analytical Chemistry (Southwest University), Ministry of Education, College of Pharmaceutical Sciences, Southwest University, Chongqing, China

**Keywords:** acute liver injury, costunolide, *Vladimiria souliei*, anti-oxidation, anti-inflammatory, anti-apoptosis

## Abstract

Costunolide, a sesquiterpene isolated from *Vladimiria souliei* (Franch.) Ling, is known to exhibit anti-inflammatory, anti-viral, and anti-tumor activities. However, the effects of costunolide on liver injury are poorly understood. The current study aimed to investigate the hepatoprotective effects of costunolide against lipopolysaccharide (LPS) and D-galactosamine-induced acute liver injury (ALI) in mice. The results indicated that costunolide (40 mg/kg) could significantly improve the pathological changes of hepatic tissue, and reduced the LPS and D-galactosamine-induced increases of alanine aminotransferase (from 887.24 ± 21.72 to 121.67 ± 6.56 IU/L) and aspartate aminotransferase (from 891.01 ± 45.24 to 199.94 ± 11.53 IU/L) activities in serum. Further research indicated that costunolide significantly reduced malondialdehyde content (from 24.56 ± 1.39 to 9.17 ± 0.25 nmol/ml) and reactive oxygen species (from 203.34 ± 7.68 to 144.23 ± 7.12%), increased the activity of anti-oxidant enzymes superoxide dismutase (from 153.74 ± 10.33 to 262.27 ± 8.39 U/ml), catalase (from 6.12 ± 0.30 to 12.44 ± 0.57 U/ml), and total anti-oxidant capacity (from 0.64 ± 0.06 to 6.29 ± 0.11 U/ml) in hepatic tissues. Western blot results revealed that costunolide may trigger the anti-oxidative defense system by inhibiting kelch-like ECH-associated protein 1 and nuclear factor-related factor 2 (cytosol), increasing nuclear factor-related factor 2 (nucleus), heme oxygenase-1 and NAD (P) H quinone oxidoreductase 1 activity. Moreover, costunolide significantly decreased the protein expression of proinflammatory cytokines including interleukin 1β, interleukin 6, and tumor necrosis factor. Pretreatment with costunolide could reduce the expression of toll-like receptor 4, myeloid differentiation factor 88, p65 (Nucleus), phosphorylated IκB kinase α/β, inhibitor of nuclear factor kappa-B kinase, inhibitor kappa Bα and prevent the expression of phosphorylated inhibitor kappa B kinase which repressed translocation of p65 from cytoplasm to nucleus. In addition, pretreatment with costunolide also inhibited hepatocyte apoptosis by reducing the expression of B-cell lymphoma 2 associated X, cytochrome C, cysteinyl aspartate specific proteinase 3, cysteinyl aspartate specific proteinase 8 and cysteinyl aspartate specific proteinase 9, and by increasing B-cell lymphoma 2. From the above analysis, the protective effects of costunolide against LPS and D-galactosamine-induced ALI in mice may be attributed to its anti-oxidative activity in nuclear factor-related factor 2 signaling pathways, anti-inflammatory suppression in nuclear factor-kappa B signaling pathways, and inhibition of hepatocyte apoptosis. Thus, costunolide may be a potential therapeutic agent in attenuating LPS and D-galactosamine -induced ALI in the future.

## Graphical Abstract

The graphical abstract on mechanism of the hepatoprotective effect of cos against ALI.

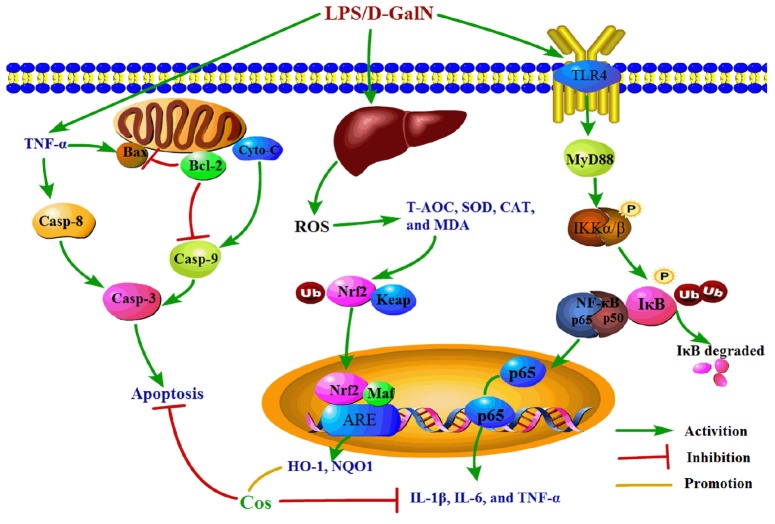

## Introduction

Acute liver injury is considered as a life-threatening complex syndrome with a variety of clinical manifestations, characterized by rapid “loss of hepatocyte function” in patients without pre-existing liver disease (Hansen et al., 2014). Without immediate control and treatment, the progression of disease may be accelerated, progressively leading to liver fibrosis, cirrhosis, and even liver failure which may require liver transplantation (Russo et al., 2004). In spite of the high mortality, there is still lack of reliable and effective drugs against liver injury. Currently, the main drug for the treatment of liver injury is silymarin. However, the combination use of sily and other drugs, such as nifedipine, irinotecan, or metronidazole, may induce therapeutic failure or increased toxicity (Wu et al., 2009). Therefore, the development of novel promising hepatoprotective agents for clinical treatment of ALI is critical.

Lipopolysaccharide (LPS) and D-galactosamine (D-GalN)-induced ALI is a well-established experimental model and its symptoms are similar to human hepatitis in the clinic. Hence it is widely used to evaluate the hepatoprotective effect, particularly regarding the extent of inflammation, apoptosis, and oxidative damage (Shin et al., 2011). D-GalN may cause hepatocytes apoptosis and necrosis in mice by depletion of uridine triphosphate in liver resulting in suppression of RNA synthesis (Wu et al., 2014). LPS is a component of the *Escherichia coli* outer membrane that may cause a typical inflammatory response mainly via TLR4-NF-κB signaling pathway (Akira and Takeda, 2004). LPS can activate Kupffer cells and macrophages to synthesize and secrete pro-inflammatory factors such as IL-6, IL-1β, and TNF-α (Das et al., 2012). Activated KCs in turn produce a large amount of ROS, pro-inflammatory cytokines, and chemokines and induce the infiltration of other inflammatory cells. The combination of D-GalN and LPS can be used to promote hepatocyte death and the development of ALI. The ROS, pro-inflammatory cytokines, and the infiltration of other inflammatory cells finally cause ALI. In present study, the murine ALI model was established by intraperitoneal injection of LPS/D-GalN.

**FIGURE 1:**
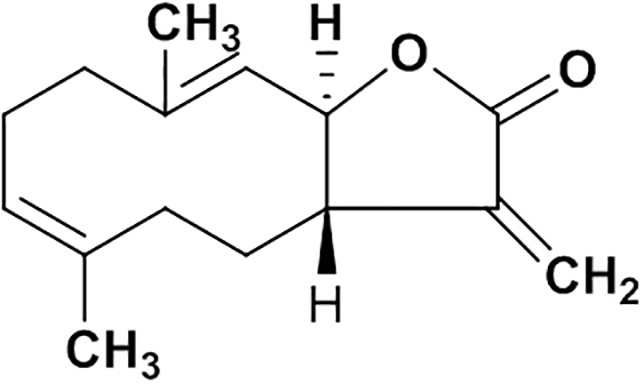
The structure of costunolide.

Natural products are important sources of novel anti-hepatitis drugs for treatment of inflammatory liver diseases (Ling et al., 2012). Plants of *Vladimiria souliei* (family Compositae) Ling, are mainly distributed in western Sichuan and eastern Tibet of China. Roots of *Vladimiria souliei* (Franch.) Ling have been used as a Traditional Chinese Medicine to alleviate abdominal pain, vomiting, borborygmus, and diarrhea for centuries (Chinese Pharmacopoeia Commission, 2015). Previous phytochemical investigations found that sesquiterpene lactones, lignans, and volatile oil are the main constituents of *Vladimiria souliei* (Franch.) Ling (Tan et al., 1990a). Especially, sesquiterpene lactones of costunolide (cos, Figure 1) and dehydrocostuslactone (Supplementary Figure S4) have been considered as the major active compounds (Tan et al., 1990b; Butturini et al., 2014). Cos has been found to exhibit diverse biological active effects such as anti-hepatitis B virus (Chen et al., 1995), inhibiting ethanol-induced gastric ulcer in mice (Zheng et al., 2016), anti-lung injury activity (Butturini et al., 2014) and anti-tumor activity (Zhang et al., 2016).

Recently, it was reported that cos also showed protective effects against LPS/D-GalN-induced liver injury in mice, which could be a promising therapeutic reagent for treatment of ALI (Wang et al., 2017). However, mechanistically only NF-κB pathway was studied. As mentioned above, the therapeutic mechanism of LPS/D-GalN inducing ALI is not only influenced by NF-κB signaling, but also associated with oxidative stress signaling pathway and apoptosis signaling pathways. Therefore, in present study, we evaluated the protective effect of cos aganist LPS/D-GalN-induced ALI and then systemically investigated the underlying mechanism through anti-oxidative, anti-inflammatory, and anti-apoptosis capabilities.

## Materials and Methods

### Materials and Reagents

The roots of *Vladimiria souliei* (Franch.) Ling were collected from Luding, Sichuan Province in September of 2015. This plant was identified by Professor Min Chen at Southwest University College of Pharmaceutical Sciences. A voucher specimen (No. CM2015-002) was deposited at the Southwest University College of Pharmaceutical Sciences (Chongqing, China).

LPS (Cat. No. L5293) was obtained from Sigma-Aldrich China, Co., LLC (Shanghai, China). D-GalN was purchased from Aladdin Reagent Database, Inc. (Shanghai, China). Diagnostic kits used for the determination of MDA, CAT, SOD, T-AOC. AST, ALT activities and cytochrome C (Cyto-C) assay kit were obtained from Nanjing Jiancheng Bioengineering Institute (Nanjing, China). Rabbit IL-6, IL-1β, IκB, p-IκB, IκB, p-IKK, NF-κB, Bax, Bcl-2, Cyto-C, Caspase3, Caspase8, Caspase9, β-actin, mouse TNF-α polyclonal primary antibodies, goat anti-mouse IgG-HRP-conjugated secondary antibody, and goat anti-rabbit IgGHRP-conjugated secondary antibody were obtained from Proteintech Group, Inc. (Wuhan, China), while total protein extraction kit was from Sangon Biotech, Co., Ltd. (Shanghai, China).

### Extraction and Isolation

Powder of the air-dried roots (11.0 kg) of *Vladimiria souliei* (Franch.) Ling was extracted by maceration with 95% ethanol overnight at room temperature. The ethanol extract was evaporated *in vacuo* to yield a semisolid (1.12 kg), which was suspended in water (5 L) and partitioned with petroleum ether (25 L), ethyl acetate (25 L), and *n*-butanol (25 L), successively. The ethyl acetate solution was concentrated to yield 296 g of residue, which was subjected to silica gel chromatography (100∼200 meshes, 70 cm × 10 cm. I.D) and eluted with petroleum ether-ethyl acetate mixtures of increasing polarity (99:1 to 10:1) to obtain total 16 fractions (A to P). They are Fr.A∼C (99:1, 12 L), Fr.D∼G (98:2, 12 L), Fr.H (95:5, 12 L), Fr.I∼J (9:1, 15 L), Fr.K (8:2, 15 L), Fr.L (7:3, 15 L), Fr.M (6:4, 15 L), Fr.N (5:5, 15 L), Fr.O (3:7, 15 L), and Fr.P (10:1, 15 L). Cos(3aS,6E,10E,11aR)-3a,4,5,8,9,11a-Hexahydro-6,10-dimethyl-3-methy lenecyclodeca[b]furan-2(3H)-one (1.35 g) was crystallized and recrystallized from fractions D∼G (98:2) with the purity above 99% (Chemical property of costunolide was shown in Supplementary Figure S1; purity data in Supplementary Figures S1, S2), which was identified by spectroscopic method (^1^H-NMR and MS, Supplementary Figures S2, S3) by comparing with literature data (Tan et al., 1990a; Cao et al., 2016).

### Animals

**FIGURE 2:**
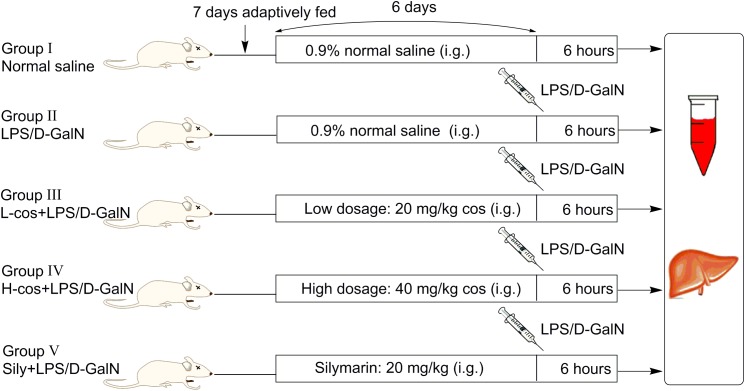
The diagrammatic of mice experiment.

A total of 50 male Kunming mice weighing (18–22 g) were purchased from Academy of Traditional Chinese Medicine of Chongqing. Before the experiment, all the mice were adaptively fed for consecutive 7 days, the diagrammatic of mice experiment was presented in Figure 2. Mice were housed in a specified chamber with controlled air conditions (temperature 20–25°C, humidity 50–65%) and free access to sterile food and water. Cos (20 and 40 mg/kg) and sily (20 mg/kg) were dissolved in normal saline respectively and intragastric (i.g) administered (gavage needle, 26G × 25 mm, 1.5 mm tip) each day for consecutive 6 days before the mice received an intraperitoneal injection with LPS/D-GalN treatment (GalN: 400 mg/kg, LPS: 50 μg/kg). Control group and LPS/D-GalN group received an equivalent volume (500 μl) of normal saline as the vehicle. Animals were randomly divided into five groups (*n* = 10/group):

(I) Control group: normal saline for consecutive 6 days;
(II) LPS/D-GalN group: mice were treated with normal saline for consecutive 6 days, then intraperitoneally injected with LPS/D-GalN;
  L-cos
(III) +LPS/D-GalN group: mice were treated with cos at 20 mg/kg body weight/day and normal saline for consecutive 6 days, then intraperitoneally injected with LPS/D-GalN;
  H-cos
(IV) +LPS/D-GalN group: mice were treated with cos at 40 mg/kg body weight/day and normal saline for consecutive 6 days, then intraperitoneally injected with LPS/D-GalN;
(V) Sily+LPS/D-GalN group: mice were treated with sily at 20 mg/kg body weight/day and normal saline for consecutive 6 days, then intraperitoneally injected with LPS/D-GalN.

Mice were anesthetized with ether, then sacrificed at 6 h after LPS/D-GalN injection, blood and liver samples were collected.

### Histopathological Examination

**FIGURE 3:**
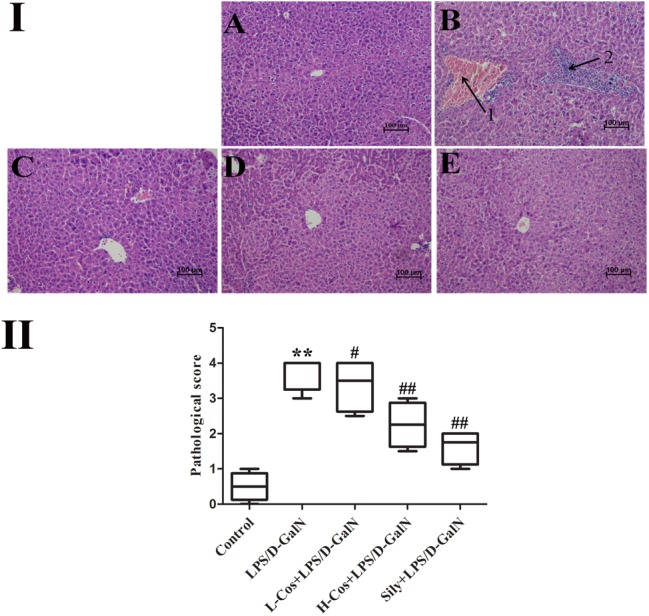
Effects of cos on liver histology in mice. **(I)** The histopathological changes of liver. **(I-A)** Control, **(I-B)** LPS/D-GalN (LPS 50 μg/kg; D-GalN: 400 mg/kg; arrow 1: central venous hyperemia; arrow 2: inflammatory infiltration), **(I-C)** LPS/D-GalN + L-cos (**I-B** + cos 20 mg/kg), **(I-D)** LPS/D-GalN + H-cos (**I-B** + cos 40 mg/kg), **(I-E)** LPS/D-GalN + sily (**I-B** + cos 40 mg/kg) (magnification: 200×). **(II)** The scores of the liver injury in the mice after treated with cos. The data represented as mean ± SD (*n* = 10). ^∗∗^*P* < 0.01 vs. Control group; ^#^*P* < 0.05, ^##^*P* < 0.01 vs. LPS/D-GalN group.

After LPS/D-GalN administration, all the mice were executed at 6 h. Hepatic tissues were collected and fixed in 10% neutral buffered formalin solution for histopathological examination. Sections (4 μm thick) were cut and stained with hematoxylin and eosin (H&E) for general pathological examination. The pathological changes of hepatic tissues were observed by a microscope and photographed at 200× magnification (Figure 3I). Based on the severity of the liver injury (hyperemia in central vein and hepatic sinusoid, hepatic portal edema, inflammatory cell infiltration, and necrosis of liver cells), the evaluation criterion of hepatic tissue was scored as follows: 0 point: very slight or no pathological change, 1 point: mild condition, 2 points: moderate condition, 3 points: severe condition, 4 points: extremely severe condition. 10 pathological section of liver tissues were collected and scored as above for each group separately (Figure 3II).

### Liver Function Assay

Six hours after administration of LPS/D-GalN, serum was acquired by centrifugation (5000 rpm, 10 min) of all blood samples. The serum activities of ALT and AST were measured using the assay kits following the manufacturer’s instruction (Nanjing Jiancheng Bioengineering Institute, Nanjing, China).

### Evaluation of Anti-oxidation Degree

Hepatic tissues were homogenized at 4°C, centrifuged at 5000 rpm for 10 min, and supernatants collected. The concentrations of SOD, MDA, CAT, and T-AOC, in diverse serum samples were tested by use of assay kits (Nanjing Jiancheng Biotechnology Institute, Nanjing, China). In addition, liver tissues were treated as cell suspension by MediMachine II single cell suspension preparation system. DCFH-DA was used as a fluorescent probe to determine the level of ROS. The fluorescence was measured on a Hitachi F7000 fluorescence spectrophotometer with an excitation wavelength was 480 nm and the emission wavelength was 538 nm.

### Immunohistochemistry Staining Assay

For immunohistochemistry, the following steps were performed as per the instructions in the Histostain^TM^-Plus and DaB substrate kits. Paraffin-embedded liver tissues were deparaffined, and sections (4 μm thick) were placed onto glass slides and incubated in blocking buffer for 30 min at room temperature. The sections were incubated at 4°C with NF-κB antibody overnight and then with biotin-labeled goat anti-rabbit IgG-HRP for 1 h at room temperature. 3, 3′-DAB solution was used for color development and specimens were counterstained with hematoxylin. The images were captured and photographed at 200× magnification by a microscope.

### Western Blot Assay

Western blot assay was performed to observe the protein expression of the related signaling pathways in livers. The liver tissue lysate was separated with 12.5% SDS-PAGE and the separated proteins transferred onto nitrocellulose membranes. Then the strip was transferred to the antibody incubating box and washed with TBST for three times. Blots were blocked in 10% skim milk at room temperature for 1.5 h and incubated overnight at 4°C with primary antibodies: IL-6 (1:5000), IL-1β (1:1000), TNF-α (1:2500), TLR4 (1:1000), MyD88 (1:500), p-IKKα/β (1:500), IKKα/β (1:500), NF-κB (1:2500), IκB (1:2500), p-IκB (1:5000), Keap-1 (1:5000), Nrf2 (1:1000), NQO1 (1:2500), HO-1 (1:1000), Bax (1:5000), Cyto-C (1:500), Bcl-2 (1:2500), Caspase3 (1:1000), Caspase8 (1:2500), Caspase9 (1:2500) Lamin B (1:5000), and β-actin (1:5000), respectively. After washing with TBST, IgG-HRP-conjugated tagged secondary antibodies (1:10000) in wash solution was added, and the solution was incubated for 1.5 h at room temperature. Target proteins were visualized and quantitated by use of Image Jet software, Lamin B or β-actin were used as an internal standards.

### Ethics Statement

Animal care, handling, sampling, and administration procedures were approved by the Southwest University Animal Care and Use Committee (Chongqing, China) prior to initiation of the experiment (Permit No. 2017-14).

### Statistical Analysis

Statistical analysis was performed using SPSS 18.0 software. The statistical significance of the differences between groups was determined by ANOVA and all the data were statistically analyzed by mean ± SD. *P* < 0.05 (5% significant level) was considered statistically significant, and *P* < 0.01 (1% significant level), *P* < 0.001 (0.1% significant level) was considered to be an extremely significant difference.

## Results

### Effects of Cos on LPS/D-GalN Induced Histopathological Changes

Histopathological analysis of the hepatic tissues was performed with H&E staining assay. The hepatic tissues in control group exhibited normal lobular architecture and cellular structure (Figure 3I-A). In contrast, LPS/D-GalN group presented portal vein inflammation and cell necrosis in a wide area, central venous hyperemia, and severe inflammatory cell infiltration (Figure 3I-B). The severity of neutrophil infiltration and other histological damage induced by LPS/D-GalN was moderately reduced in L-cos group (Figure 3I-C). However, the specimens of H-cos group presented only a mild degree of necrotic and degenerative changes with less neutrophil infiltration (Figure 3I-D), which was more similar to the healthy control group. In addition, the histological changes in LPS/D-GalN treated groups manifested in a dose dependent manner. The sily group showed clearly less necrotic and degenerative changes and less inflammatory cell infiltration (Figure 3I-E). Based on the score of evaluation standards of hepatic tissue (Figure 3II), significant pathological changes were observed. Compared with control group, the score of liver injury increased in LPS/D-GalN group (^∗∗^*P* < 0.01) and decreased in L-cos group and H-cos group (^##^*P* < 0.01).

### Effects of Cos on Serum ALT, AST

**FIGURE 4:**
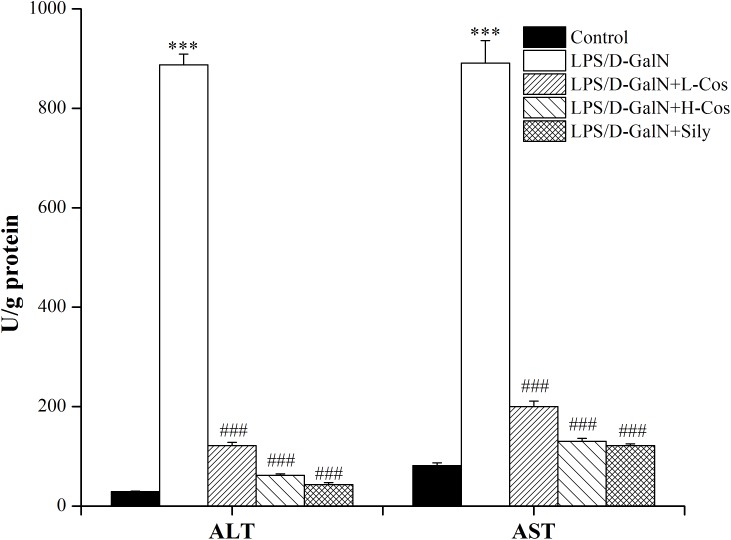
Effects of cos on the activities of AST and ALT induced by LPS/D-GalN. The data represent the mean ± SD (*n* = 10). ^∗∗∗^*P* < 0.001 vs. control group; ^###^*P* < 0.001 vs. LPS/D-GalN group.

Hepatic injury was evaluated by the activities of AST and ALT enzymes. As shown in Figure 4, a significant increase in serum ALT and AST levels was observed in LPS/D-GalN group compared with control group. In contrast, mice pretreated with cos or sily showed a significant reduction in the level of these parameters. The data further confirmed that cos plays a hepatoprotective role in the LPS/D-GalN induced ALI model.

### Effects of Cos on Lipid Peroxidation and Anti-oxidant Enzyme Activities

The quantitative analysis of MDA, SOD, CAT, T-AOC, and ROS (Table 1) were observed as indicators for the assessment of LPS/D-GalN induced oxidant damages. SOD, CAT, and T-AOC activity levels were significantly lowered after 6 h with LPS/D-GalN administration, while MDA and ROS were significantly increased. Pretreatment with cos effectively lowered MDA and ROS levels (*P* < 0.01), and significantly increased the levels on liver CAT, SOD, and T-AOC (*P* < 0.01), compared with LPS/D-GalN group. In mice with LPS/D-GalN induced ALI, cos significantly improved the content of MDA, SOD, CAT, T-AOC, and ROS to exert their beneficial effects against oxidative stress induced by hepatic injury. The results revealed that cos significantly protects against LPS/D-GalN induced ALI by reducing MDA and ROS production, and by effectively promoting CAT, SOD, and T-AOC activity.

**Table 1:** Effects of cos on the activities of hepatic SOD, MDA, CAT, T-AOC, and ROS in LPS/D-GalN treated mice.

Group	SOD (U/ml)	MDA (nmol/ml)	CAT (U/ml)	T-AOC (U/ml)	ROS (%)
Control	248.70 ± 6.95	6.83 ± 0.33	18.36 ± 0.81	4.46 ± 0.40	98.91 ± 3.43
LPS/D-GalN	153.74 ± 10.33^∗∗^	24.56 ± 1.39^∗∗^	6.12 ± 0.30^∗∗^	0.64 ± 0.06^∗∗^	205.26 ± 8.43^∗∗^
LPS/D-GalN+L-cos	160.68 ± 7.45	16.56 ± 0.77^##^	7.47 ± 0.53	2.20 ± 0.15^#^	175.40 ± 6.32^#^
LPS/D-GalN+H-cos	262.27 ± 8.39^##^	9.17 ± 0.25^##^	12.44 ± 0.57^##^	6.29 ± 0.11^##^	139.53 ± 4.18^##^
LPS/D-GalN+Sily	281.56 ± 18.60^##^	8.28 ± 0.37^##^	16.85 ± 0.38^##^	10.31 ± 0.81^##^	138.41 ± 6.97^##^


*The data represent the mean ± SD (*n* = 10). ^∗∗^*P* < 0.01 vs. control group; ^#^*P* < 0.05, ^##^*P* < 0.01 vs. LPS/D-GalN group.*

### Effects of Cos on the Expression of Pro-inflammatory Factors

**FIGURE 5:**
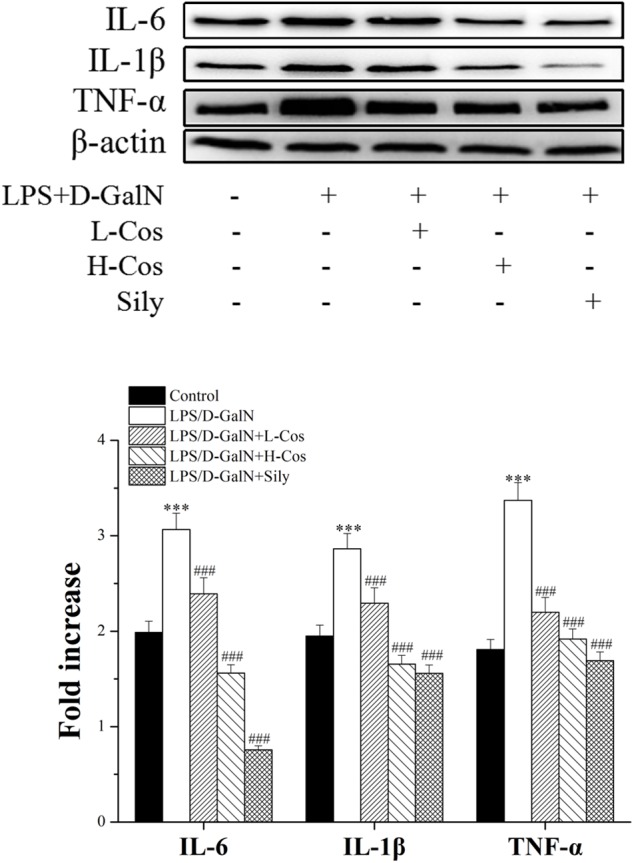
Effects of cos on the expression of TNF-α, IL-6, and IL-1β after LPS/D-GalN administration by western blot analysis. The data represent the mean ± SD (*n* = 10). ^∗∗∗^*P* < 0.001 vs. control group; ^##^*P* < 0.01 and ^###^*P* < 0.001 vs. LPS/D-GalN group.

Pro-inflammatory mediators/cytokines are known to play important roles in the pathogenesis of ALI (Li et al., 2017). The expressions of the key proteins were examined with western blotting assay. As shown in Figure 5, the levels of IL-6, IL-1β, and TNF-α were significantly increased in the LPS/D-GalN group. However, those pro-inflammatory mediators/cytokines were significantly reduced with addition of cos in a dose dependent manner indicating that cos may inhibit the production of pro-inflammatory mediators/cytokines induced by LPS/D-GalN. Thus, it was confirmed that cos plays a hepatoprotective role in the LPS/D-GalN induced ALI model via down-regulating the levels of IL-6, IL-1β, and TNF-α.

### Effects of Cos on Hepatic Activities of NF-κB Signaling Pathway

**FIGURE 6:**
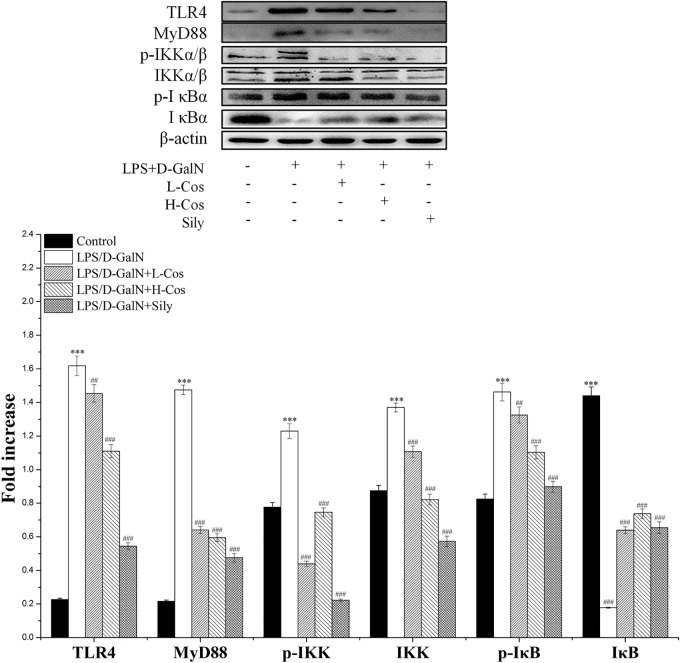
Effects of cos on the expression of NF-κB-MyD88 signaling pathway. Western blot analysis of the TLR4, MyD88, IKK, p-IKK, IκB, and p-IκB proteins. The density values of the western blot were normalized for β-actin. ^∗∗∗^*P* < 0.01 vs. control group; ^##^*P* < 0.01 and ^###^*P* < 0.0001 vs. LPS/D-GalN group.

**FIGURE 7:**
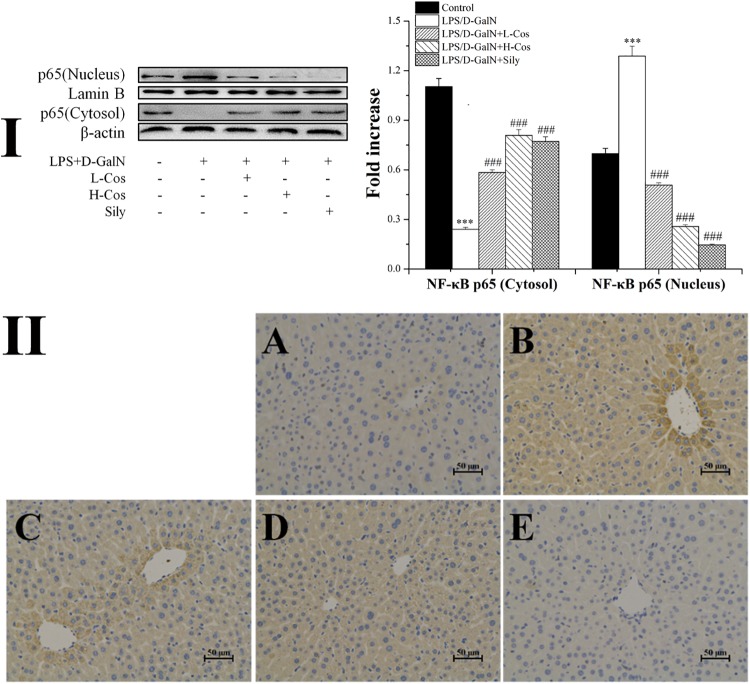
Effects of cos on the expression of p65 protein. **(I)** Western blot analysis of cytosol and nucleus p65 proteins. **(II)** Immunohistochemistry analysis of NF-κB (p65). **(II-A)** Control; **(II-B)** LPS/D-GalN (LPS 50 μg/kg; D-GalN: 400 mg/kg); **(II-C)** LPS/D-GalN + L-cos (**II-B** + cos 20 mg/kg); **(II-D)** LPS/D-GalN + H-cos (**II-B** + cos 40 mg/kg); **(II-E)** LPS/D-GalN + sily (**II-B** + sily 20 mg/kg). The density values of the western blot were normalized for β-actin and Lamin B. ^∗∗∗^*P* < 0.01 vs. control group, ^###^*P* < 0.01 vs. LPS/D-GalN group.

As shown in Figure 6, the expressions of TLR4, MyD88, p65 (Nucleus), p-IKKα/β, IKKα/β, and p-IκBα in LPS/D-GalN group were significantly increased while the levels of IκBα and p65 (Cytosol) were significantly lowered than in the control group. However, prior treatment with cos significantly enhanced the expression levels of TLR4, MyD88, p-IKKα/β, IKKα/β, and p-IκBα (*P* < 0.001) and increased level of IκBα (*P* < 0.001). The expression of p65 cytosolic protein was found to be maintained in control animals. However, the majority of p65 protein translocated from cytosol to the nucleus after LPS/D-GalN administration. Compared to LPS/D-GalN group, pretreatment of cos reduced this translocation (*P* < 0.001) (Figure 7I). The immunohistochemistry assay showed that positive staining of p65 in the H-cos, L-cos, and sily groups were less than that in the LPS/D-GalN group (Figure 7II). Further histopathological findings supported the biochemical results. Combined results of western blot and immunohistochemistry showed that cos could inhibit LPS/D-GalN-induced ALI via NF-κB signaling pathway by up-regulating the levels of TLR4, MyD88, p65 (Nucleus), p-IKKα/β, IKKα/β, and p-IκBα proteins and down-regulating the levels of p65 (Cytosol) and IκBα protein.

### Effects of Cos on the Expression of Nrf2 Signaling Pathway

**FIGURE 8:**
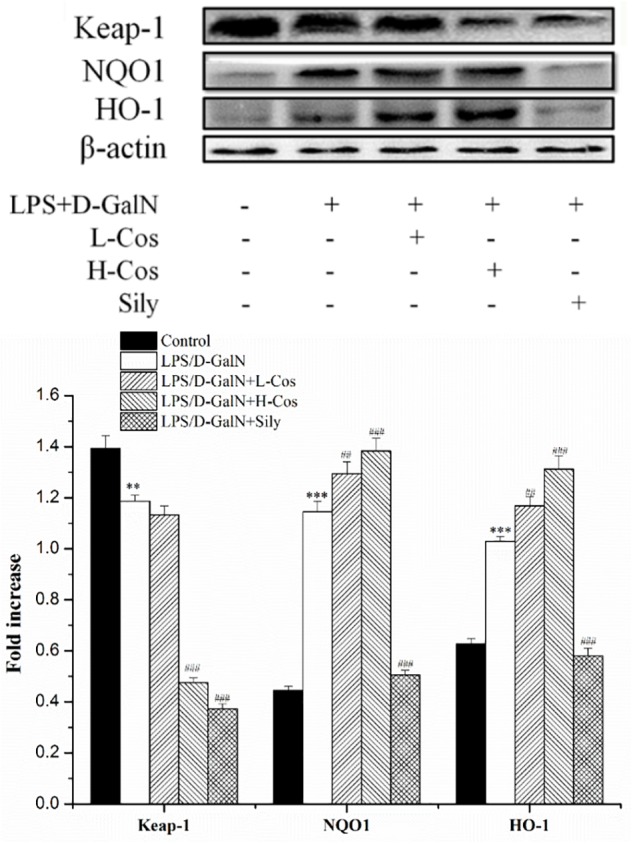
Effects of cos on the expression Nrf2 signaling pathway. The expression of Nrf2 (cytosol and nucleus) were analyzed by western blot. The data represent the mean ± SD (*n* = 10). The density values of the western blot were normalized for β-actin and Lamin B. ^∗∗^*P* < 0.01, ^∗∗∗^*P* < 0.001 vs. control group; ^###^*P* < 0.001 and ^##^*P* < 0.01 vs. LPS/D-GalN group.

**FIGURE 9:**
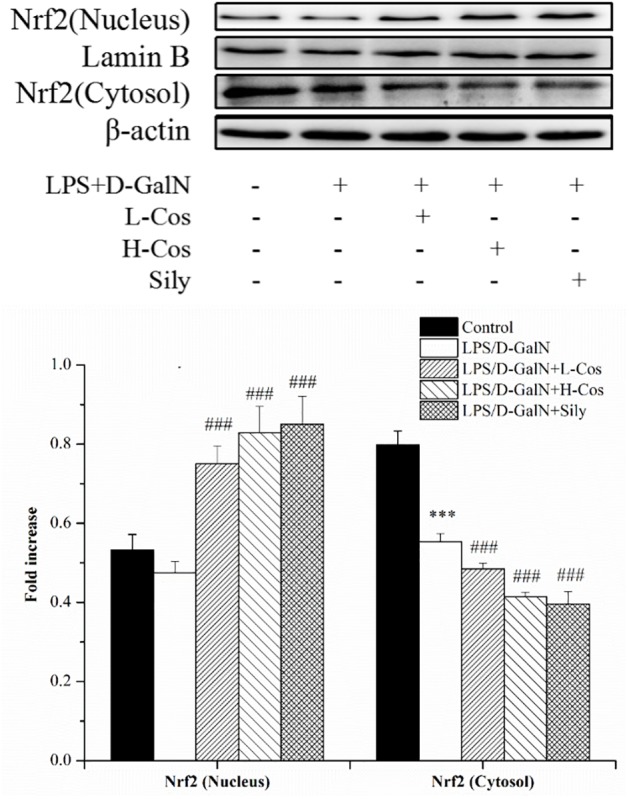
Effects of cos on the expression of proteins related to Keap-1 signaling pathway. The expression of hepatocyte oxidative stress related proteins Keap-1, HO-1, NQO1 were analyzed by western blot. The data represented as mean ± SD (*n* = 10). ^∗∗^*P* < 0.01, ^∗∗∗^*P* < 0.001 vs. control group; ^##^*P* < 0.01 and ^###^*P* < 0.001 vs. LPS/D-GalN group.

The Nrf2-Keap1 system is recognized as one of the major cellular defense mechanisms against oxidative stress to protect liver from injury (Jadeja et al., 2016). To explore the mechanism of the protective effect of cos against ALI induced by LPS/D-GalN in mice through Nrf2 signaling pathway, the expression of hepatocyte oxidative stress related proteins Keap-1, HO-1, and NQO1 were analyzed by western blot (Figure 8). In addition, the cytosol and nucleus of Nrf2 were analyzed by western blot (Figure 9). On pretreatment with cos, the levels of NQO1, HO-1, and Nrf2 (nucleus) significantly increased while the level of Keap-1 and Nrf2 (cytosol) markedly decreased (*P* < 0.001). The results showed that cos may inhibit LPS/D-GalN-induced ALI via Nrf2 signaling pathway by up-regulating the levels of NQO1, HO-1, and Nrf2 (nucleus) proteins and down-regulating the level of Keap-1 and Nrf2 (cytosol) proteins.

### Effects of Cos on the Expression of Apoptosis-Related Proteins

**FIGURE 10:**
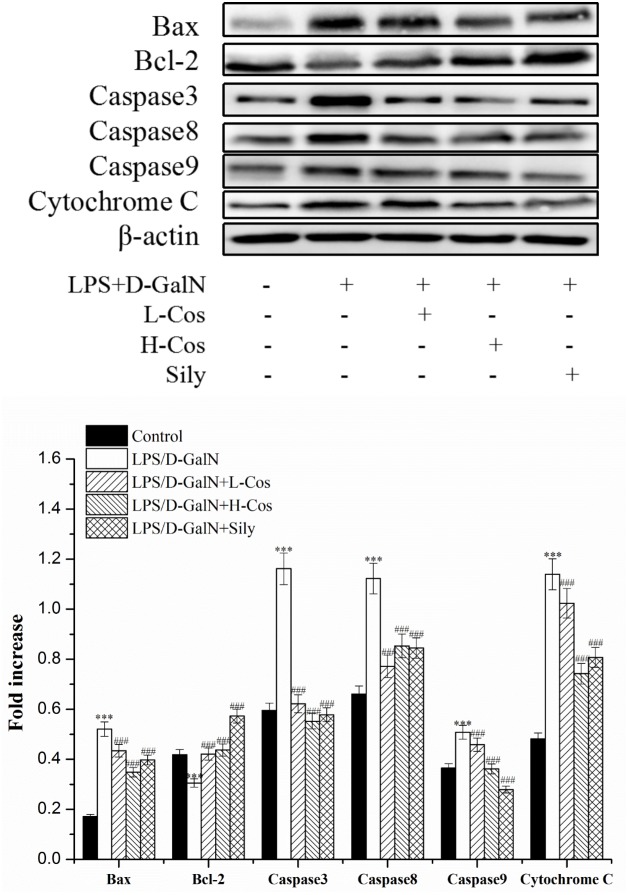
Effects of cos on the expression of apoptosis-related proteins. The expression of hepatocyte apoptosis related proteins Bax, Bcl-2, Caspase3, Caspase8, and Caspase9 were analyzed by western blot. The data represent the mean ± SD (*n* = 10). ^∗∗∗^*P* < 0.001 vs. control group; ^###^*P* < 0.001 and ^##^*P* < 0.01 vs. LPS/D-GalN group.

Apoptosis occurs in the process of liver injury that leads liver cells to death (Bantel and Schulze-Osthoff, 2012). To investigate the inhibitory effect of cos on hepatocyte apoptosis induced by LPS/D-GalN, apoptosis related signal transduction proteins (Bcl-2, Bax, Cyto-C, Caspase3, Caspase8, and Caspase9) were detected by western blotting assay (Figure 10). Compared with control group, the levels of these proteins significantly increased (*P* < 0.001), and the level of Bcl-2 protein significantly decreased (*P* < 0.001) in LPS/D-GalN group. In contrast, after cos treatment, while the level of Bcl-2 protein significantly increased (*P* < 0.001), Bax, Cyto-C, Caspase3, Caspase8, and Caspase9 proteins were significantly decreased (*P* < 0.001). These findings suggested that cos could reverse the hepatocytes apoptosis induced by LPS/D-GalN by up-regulating the level of Bcl-2 protein and down-regulating the levels of Bax, Cyto-C, Caspase3, Caspase8, and Caspase9 proteins.

## Discussion

Alanine aminotransferase and AST are two important biochemical indicators for the diagnosis of liver injury in clinical trials. When the liver cells were damaged by LPS/D-GalN induced ALI, the cell membrane was broken and the cells appeared to be degenerate and necrotic. ALT and AST which existed in the cells were released into the blood, and the levels of ALT and AST in the blood finally increased (Khattab et al., 2015). In present study, cos significantly reduced serum ALT and AST levels on LPS/D-GalN-induced ALI. There was high mortality, inflammatory infiltration, necrosis of liver cells, bleeding, and loss of hepatic architectures after LPS/D-GalN administration. However, these phenomena were hindered by pretreatment with cos. The results showed that cos alleviated the histopathological changes of liver injury and significantly reduced serum ALT and AST levels indicating cos may protect against LPS/D-GalN induced ALI.

The imbalance between pro- and anti-inflammatory cytokines is a hallmark of the pathogenesis of ALI. Previous studies showed that hepatic injury is associated with the production of pro-inflammatory cytokines (IL-6, IL-1β, and TNF-α) in the pathogenesis of ALI (Pastora et al., 1995; Li et al., 2017). IL-6 is a pleiotropic cytokine produced in response to inflammatory stimuli. TNF-α exhibits both the proinflammatory and immunoregulatory properties of cytokines. IL-1β as a member of IL-1 cytokine family, is a pleiotropic and immunoregulatory cytokine. It has been demonstrated that proinflammatory cytokines such as IL-6, TNF-α, and IL-1β show altered levels in ALI mice (Lu et al., 2016). In the present study, it was found that cos significantly decreased the levels of TNF-α, IL-6, and IL-1β in LPS/D-GalN administrated mice. The production of pro-inflammatory cytokines was mainly caused by the activation of NF-κB signaling pathway. NF-κB transcription factors (p65 and p50) are critical regulators of pro-inflammatory responses, which are associated with the inhibitory IκB family proteins (IκBs) (Cildir et al., 2016). In its inactive form, NF-κB is normally sequestered in the cytoplasm by its inhibitory proteins IκBα. When stimulated by LPS, inhibitor of NF-κB kinase (IKK) is activated, which triggers the dissociation of cytoplasmic NF-κB/IκB complex (Dobrovolskaia and Vogel, 2002). The IKK-mediated phosphorylation and proteasomal degradation of IκB enables the translocation of active NF-κB transcription factor subunit (p65) into the nucleus leading to activation of the expression of pro-inflammatory mediators (IL-6, IL-1β, TNF-α) (Zhang et al., 2017). The release of pro-inflammatory cytokines activates NF-κB to amplify the pro-inflammatory response cascade, as a positive feedback. TLR4 mediated MyD88 signaling pathway plays an important role in the regulation of hepatic immune dysfunction induced by inflammatory reaction (Xu et al., 2017). TLR4 is an important protein molecule involved in immunity which can be activated by LPS. The activation of TLR4 may recruit the signaling adaptor MyD88, and the TH domain in the TLR4 intracellular domain interacts with the carboxyl terminal TH domain of MyD88 in the cytoplasm and activates MyD88. The activation of MyD88 would lead to an activation NF-κB signaling pathway and production of pro-inflammatory cytokines, which are responsible for activating the innate immune system (Kang et al., 2017). In present study, the hepatoprotective effect of cos against ALI may be associated with NF-κB signaling pathway by the down-regulation of the expression of TLR4, MyD88, p65 (nucleus), p-IKKα/β, IKKα/β, p-IκBα, and up-regulation of the expression levels of IκBα and p65 (cytosol).

Apart from NF-κB signaling pathway, the oxidative stress generated by lipid peroxidation and oxygen free radicals are also important factors causing liver injury (Caraceni et al., 1994). ROS accumulate in the body and cause oxidative damage to tissues and organs while SOD which catalyzes conversion of superoxide to oxygen and hydrogen peroxide in aerobic organisms can scavenge free radicals and inhibit free radical reactions (Wang et al., 2016). CAT converts the H_2_O_2_ generated within cells to H_2_O and O_2_ and prevents the conversion of H_2_O_2_ into more active species as ⋅OH. SOD and CAT defend against oxidative stress by catalyzing and decomposing O_2_⋅- radicals and H_2_O_2_ respectively (Castro-Alférez et al., 2017). T-AOC, a biomarker measuring the antioxidant potential of body fluids, including redox synergistic interactions in a system, serves as a preventive index of oxidant damages (Manafikhi et al., 2017). The increase in free radicals leads to excessive production of MDA, which is considered as a biomarker of lipid peroxidation. The increase of MDA has been considered a key feature in ALI (Lykkesfeldt, 2007). In present study, the levels of SOD, CAT, T-AOC were significantly reduced while that of MDA and ROS significantly increased, indicating the hepatoprotective effects of cos may be triggered also by an anti-oxidant defense system in ALI mice.

Almost all modes of ALI are associated with increased oxidative stress and an overwhelmed antioxidant defense system. Since Nrf2 activation is associated with the enhancement of endogenous antioxidant system, it can be used as an ideal therapeutic target for reducing oxidative stress. Nrf2-ARE pathway boosts hepatocyte antioxidant defense system and may be able to reduce pathogenesis of ALI (Jadeja et al., 2016). As an oxidative stress receptor, Nrf2 is involved in cell anti-oxidant stress caused by exogenous toxic substances (Chowdhry et al., 2010). Keap1 has been shown to interact with Nrf2, a master regulator of the antioxidant response, which is important for the amelioration of oxidative stress (Ogura et al., 2010). Under normal physiological conditions, Keap1 and Nrf2 are in a state of mutually stable binding. When oxidative stress occurs, Nrf2 releases from Keap1 and thus translocates from the cytoplasm into the nucleus. In the nucleus, Nrf2 interacts with antioxidation response element (ARE) to regulate the expressions of peroxiredoxins and phase II detoxification enzymes such as HO-1 and NQO-1, which protect cells from oxidative damage and inflammation (Deramaudt et al., 2013). Our present study indicated that the expression level of Nrf2 (nucleus) increased while that of Keap-1 and Nrf2 (cytosol) reduced when pretreated with cos. The capability to protect cells from oxidative challenge and the ability to reduce quinones via an obligate two electron mechanism, which precludes generation of reactive oxygen radicals, demonstrates that NQO1 is a chemoprotective enzyme (Dinkova-Kostova and Talalay, 2010). HO-1 is an antioxidant enzymes which catalyzes the degradation of heme, and the enzyme catalyzed reaction products including carbon monoxide and bilirubin have anti-oxidative damage function (Soares et al., 2009). In present study, the levels of Nrf2 (nucleus), HO-1 and NQO1 increased significantly, while the level of Keap-1 and Nrf2 (cytosol) reduced significantly, indicating that the mechanism of the hepatopretective effect of cos against ALI may be achieved via the Nrf2 signaling pathway.

Apoptosis is a pivotal step which is considered as one of the main mechanisms of cell death in liver injury diseases (Wang, 2015). Therefore, the related apoptotic proteins (Bcl-2, Bax, Cyto-C, Caspase3, Caspase8, and Caspase9) were studied by western blotting assay. During Apoptosis, the Cyto-C of the mitochondrial membrane space is released to the cytoplasm and triggers the caspase cascade, which leads to cell death (Kluck et al., 1997). Caspase family proteins also play a key role in inducing apoptosis which are the ultimate pathway for the activation and execution of apoptosis. Among them, Caspase3, Caspase8, and Caspase9 are at the nexus of critical regulatory networks controlling cell apoptosis and death inflammation. Caspase-8 is the chief executor of apoptosis, leading to the release of Cyto-C, thus triggering apoptosis in the intracellular pathway (Salvesen, 1999). Caspase-3 is the key protease of apoptosis. When it is activated, the cascade reaction of downstream apoptosis is inevitable. The activation of Caspase-3 is closely related to the apoptosis of liver cells and eventually leads to liver injury (Ko et al., 2017). Caspase-9, as an essential initiator caspase required for apoptosis signaling through the mitochondrial pathway, is activated on the apoptosome complex (Wurstle et al., 2012). Bcl-2 is an important protein on the mitochondrial pathway that prevents the release of apoptotic protein Cyto-C into the cytoplasm by stabilizing the membrane potential of mitochondria and maintaining the integrity of mitochondria (Kluck et al., 1997; Wu et al., 2017). However, Bax facilitates the release of Cyto-C, inducing cell apoptosis. When the body is stimulated by inflammation and oxidation, TNF-α can be activated by binding to the tumor necrosis factor receptor L (TNF-R1) followed by activation of the Caspase8. Then the pro-apoptotic protein Bax is activated and the anti-apoptotic protein Bcl-2 is inhibited, which further activates Caspase3 and Caspase9, and finally leads to apoptosis of hepatocytes (Wang, 2014). In present study, we observed that cos exhibits the hepatoprotective effects on LPS/D-GalN-induced ALI mice by up-regulating the level of Bcl-2 protein and down-regulating the levels of Bax, Cyto-C, Caspase3, Caspase8, and Caspase9 proteins. It was indicated that one of the mechanism of the hepatopretective effect of cos against ALI may be achieved via the apoptosis signaling pathway.

Taken together, the underlying mechanism of the hepatopretective effect of cos against ALI was mainly connected with suppressing the production of pro-inflammatory mediator/cytokines, suppressing oxidative stress, and attenuating hepatocyte apoptosis through Nrf2, NF-κB and apoptosis pathway, respectively. The mechanism of the hepatoprotective effect of cos against ALI can be summarized briefly from the Graphical Abstract. Therefore, the TLR4-MyD88-NF-κB (p65), Keap1-Nrf2/HO-1 and apoptosis signal pathway maybe involved in the process of hepatopretective against ALI (graphical abstract).

## Conclusion

The present study demonstrated that cos exhibited potent protective effect against ALI in mice induced by LPS/D-GalN. And the protective mechanism might be connected with enhancing the anti-oxidative defense system, suppressing the inflammatory response and preventing hepatocyte apoptosis. In conclusion, our experimental evidence suggested that cos can be used as an effective candidate drug for the treatment of ALI.

## Author Contributions

MC conceived and designed the research. JM performed most of the experiments and wrote the paper. RW and MY performed parts of the experiments. YH analyzed the data.

## Conflict of Interest Statement

The authors declare that the research was conducted in the absence of any commercial or financial relationships that could be construed as a potential conflict of interest. The reviewer HZ declared a past co-authorship with one of the authors MC to the handling Editor.
