# Supplementary material for: Protective Effects of Costunolide Against D-Galactosamine and Lipopolysaccharide-Induced Acute Liver Injury in Mice
Source: Front Pharmacol. 2018 Dec 20;9:1469. doi: 10.3389/fphar.2018.01469 (PMC6307542; doi:10.3389/fphar.2018.01469)
Supplement: Supplementary file 1 [file Data_Sheet_1.docx]

**Protective Effects of Costunolide against D-Galactosamine and Lipopolysaccharide-Induced Acute Liver Injury in Mice.**

**Jingxin Mao^a^, Man Yi^a^, Rui Wang^a^, Yuanshe Huang^a^ and Min Chen^a*^**

^a^ *College of Pharmaceutical Sciences, Key Laboratory of Luminescent and Real-Time Analytical Chemistry (Southwest University), Ministry of Education, Southwest University, Chongqing 400715, P.R. China*

Running title: Hepatoprotective effects of costunolide

*Corresponding author

Prof. Min Chen

College of Pharmaceutical Sciences, Southwest University, Chongqing, P.R. China.

E-mail: mminchen@swu.edu.cn

Phone: +86 23 68250579.

Fax: +86 23 68251225.

**List of supporting information**

S1 Chemical property of costunolide.

S2. Purity Analysis of costunolide.

Figure S1. Purity Analysis of costunolide.

Figure S2. The ^1^H-NMR spectrum of costunolide.

Figure S3. The Mass spectrum of costunolide.

Figure S4. The structure of dehydrocostuslactone.

S1 Chemical property of costunolide.

**ACS of Costunolide: 553-21-9**

Costunolide, colorless needle crystal (CHCl_3_, ESI-MS m/z : 231.0 [M-H]^-^), melting point: 106-107℃. ^1^H-NMR (CDCl_3_, 400MHz): δ: 6.26 (1H, d, J=3.6Hz, H-13a), 5.52(1H, d, J=3.2Hz, H-13b), 4.85 (1H, dd, J=10.5, 1.6Hz, H-1), 4.74 (1H, d, J=1.4Hz, H-5), 4.57 (1H, t, J=8.7Hz. H-6), 1.70(3H, s, H-15), l.42 (3H, s, H-14). ^13^C-NMR(CDCl_3_, 400MHz): δ: 170.2(C-12), 141.7(C-11), 141.5(C-4), 138.1(C-10), 129.8(C-5), 127.8(C-1), 119.1(C-13), 82.1(C-6), 51.0(C-7), 41.6(C-3), 39.8(C-9), 28.7(C-2), 26.7(C-8), 17.2(C-15), 16.1(C-14).

S2 Purity analysis of costunolide

Shimadzu LC-20A high performance liquid chromatography with dual solvent pump high-pressure gradient system, SPD-20A photodiode array detector, and an autosampler were used for the first-dimension separation.

Standards and samples were accurately weighed and dissolved in methanol respectively, and the costunolide and standard sample with the final concentrations at was 1.0 mg/mL were prepared and stored at 4 ℃ before analysis. The samples were filtered with 0.45 μm organic membrane to prepare for HPLC analysis. Chromatographic elution was conducted with binary mobile phase gradient consisting of water (A) and methanol (B) in both solvents. Initial gradient conditions were set to 5% B at the ﬂow rate of 1.0 mL/min, gradient increasing to 100% B over 60 min. The column temperature was maintained at 40 ℃ during the whole process. Analysis chromatographic column Shimadzu C18 (250mm × 4.6mm, 5μm) was used and the detection wavelength is 254 nm. The volume of sample and standards injection are both 20 μL. The retention time of sample and standards are 25.32 min and 25.52 min respectively.

Using the external standard method to determine the purity of costunolide by HPLC. Samples were analyzed qualitatively using relative reservation index, and analyzed quantitatively by area normalization. According to the formula: peak area of sample /peak area of standard = concentration of sample /concentration of standard. The actual concentration of sample is 0.9904 mg/ml. And the purity was calculated by the theoretical concentration divided by the actual concentration of sample. As a result, the purity of costunolide which derives from *Vladimiria souliei (Franch.)* Ling is determined to be 99.04%.

A:

B:

Figure S1. Purity analysis of costunolide.

A: The HPLC of costunolide standards which was purchased from Sigma-Aldrich China Co., LLC. (Shanghai, China); B: The HPLC of costunolide which isolated from *Vladimiria souliei* (Franch.) Ling.

**
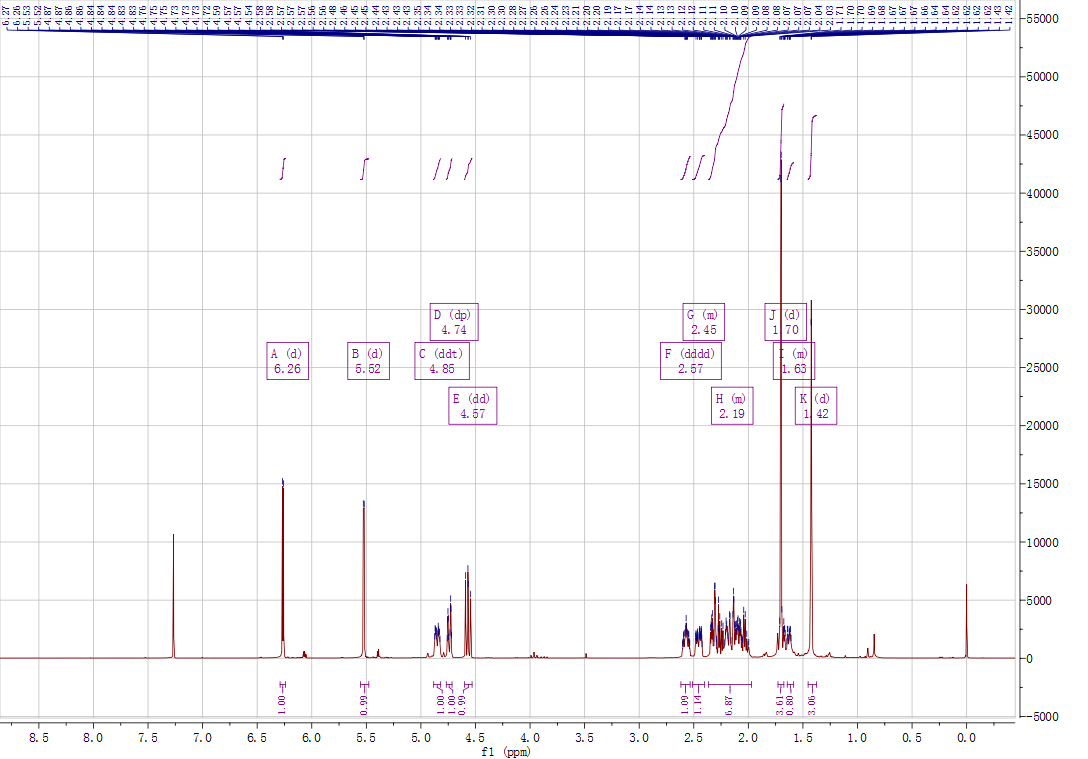
**

Figure S2. The ^1^H-NMR spectrum of costunolide.


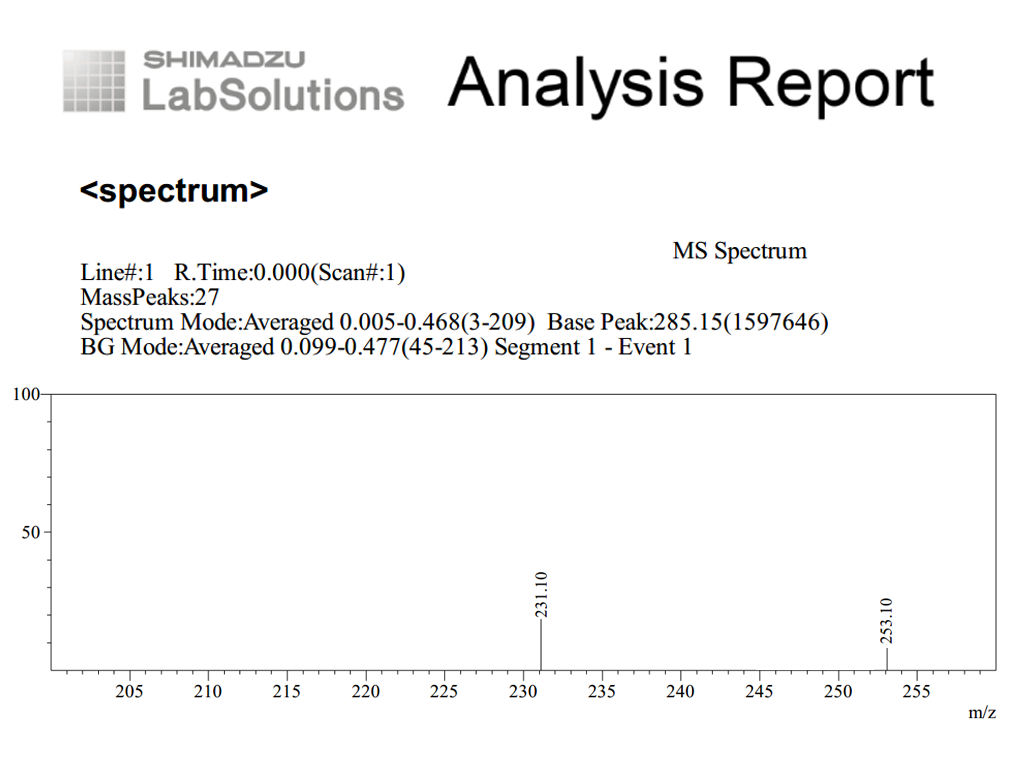


Figure S3. The Mass spectrum of costunolide.

Figure S4. The structure of dehydrocostus lactone.
